# Machine learning framework to extract the biomarker potential of plasma IgG N-glycans towards disease risk stratification

**DOI:** 10.1016/j.csbj.2024.03.008

**Published:** 2024-03-11

**Authors:** Konstantinos Flevaris, Joseph Davies, Shoh Nakai, Frano Vučković, Gordan Lauc, Malcolm G. Dunlop, Cleo Kontoravdi

**Affiliations:** a Department of Chemical Engineering, Imperial College London, London SW7 2AZ, United Kingdom; b Genos Glycoscience Research Laboratory, Zagreb 10000, Croatia; c Department of Biochemistry and Molecular Biology, Faculty of Pharmacy and Biochemistry, University of Zagreb, Zagreb, Croatia; d Colon Cancer Genetics Group, Institute of Genetics and Cancer, Cancer Research UK Scotland Centre, University of Edinburgh and Medical Research Council Human Genetics Unit, Edinburgh, United Kingdom

**Keywords:** Glycosylation, Cancer, Multi-objective optimization, Probability calibration, Interpretable machine learning

## Abstract

Effective management of chronic diseases and cancer can greatly benefit from disease-specific biomarkers that enable informative screening and timely diagnosis. IgG N-glycans found in human plasma have the potential to be minimally invasive disease-specific biomarkers for all stages of disease development due to their plasticity in response to various genetic and environmental stimuli. Data analysis and machine learning (ML) approaches can assist in harnessing the potential of IgG glycomics towards biomarker discovery and the development of reliable predictive tools for disease screening. This study proposes an ML-based N-glycomic analysis framework that can be employed to build, optimise, and evaluate multiple ML pipelines to stratify patients based on disease risk in an interpretable manner. To design and test this framework, a published colorectal cancer (CRC) dataset from the Study of Colorectal Cancer in Scotland (SOCCS) cohort (1999–2006) was used. In particular, among the different pipelines tested, an XGBoost-based ML pipeline, which was tuned using multi-objective optimisation, calibrated using an inductive Venn-Abers predictor (IVAP), and evaluated via a nested cross-validation (NCV) scheme, achieved a mean area under the Receiver Operating Characteristic Curve (AUC-ROC) of 0.771 when classifying between age-, and sex-matched healthy controls and CRC patients. This performance suggests the potential of using the relative abundance of IgG N-glycans to define populations at elevated CRC risk who merit investigation or surveillance. Finally, the IgG N-glycans that highly impact CRC classification decisions were identified using a global model-agnostic interpretability technique, namely Accumulated Local Effects (ALE). We envision that open-source computational frameworks, such as the one presented herein, will be useful in supporting the translation of glycan-based biomarkers into clinical applications.

## Graphical Abstract

**Figure:**
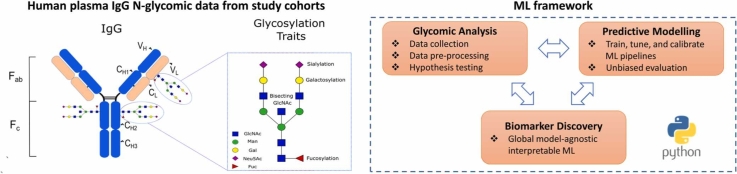

## 1 Introduction

In primary care, disease diagnosis heavily relies on the comprehensive assessment of the patient’s medical history and current symptoms, which may often be non-specific and only poorly predict the presence of disease. This degree of uncertainty in diagnosis, even for common disease conditions, leads to high estimated false negative diagnostic errors, thus hindering effective and timely interventions [1]. False positives also present a significant challenge due to strains posed on diagnostic resources. Among diseases such as cancer, traditionally implemented diagnostic procedures also have limitations regarding accessibility to patients and invasiveness [2]. Imaging technologies offer valuable insight into the tumour’s size and position [3], but they may also be less accessible to patients in low- and middle-income countries [4]. Endoscopy and tissue biopsies are effective yet invasive diagnostic tools [5]. These considerations point to a need for the identification of reliable, minimally invasive disease-specific biomarkers for diagnosis along with risk analysis tools.

Glycomics is a subfield of molecular biology concerned with the study of the glycome that is generally underrepresented compared to other omics [6], [7], but has great potential to advance biomarker research in the context of chronic inflammatory diseases and cancer [8], [9], [10]. Glycans are structurally diverse natural biopolymers of monosaccharides considered to be directly implicated in every major disease pathophysiology [11]. Glycomic profiles depend on the expression and activity of glycan-modifying enzymes and the metabolome of organisms under different temporal conditions and physiological states [12]. Although the functional role of glycans in diseases such as cancer has not yet been fully characterised, significant progress has been made in identifying associations between cancer manifestation and aberrant glycosylation profiles [13], [14]. To this end, immunoglobulin G (IgG) and its N-glycans have been of interest due to a multitude of factors. IgG plays a central role in adaptive immunity, accounts for 10–20% of the total plasma proteome, thus facilitating analytical detection [15], and has a long half-life of approximately 12 days, making it suitable for detecting chronic inflammation [16]. N-linked glycomic profiles have been shown to be temporally stable in healthy individuals [17] and the analytical measurement of IgG N-glycans is more mature compared to that of other proteins and/or glycosylation types [18]. This can be attributed to the well-characterised N-linked glycosylation sites at its crystallisable fragment (Fc) [19], the commercial interest in biomanufacturing IgG-based monoclonal antibodies with desired glycosylation profiles [20], and the emergence of high-throughput techniques [18]. Given the extensive research on the link between IgG glycosylation and pathophysiology, changes in the former appear to be sensitive to perturbations occurring in many health states, ranging from initial symptom manifestation to phenotypes preceding severe disease [13], [21]. These findings consistently point to the plasticity of IgG glycans with respect to various stimuli, making it a unique indicator of health and disease [22].

Identification of differentially abundant glycan structures and/or groups of glycan structures sharing similar structural properties (i.e., derived traits) in case-control studies is generally achieved via statistical hypothesis testing and/or multiple regression analysis [13], [23]. Given the varying cohort size of these studies, which can range from a few tens to a few thousands and the challenges of performing glycomic analysis, a small number of studies has attempted to build predictive models for disease classification using IgG N-glycan relative abundance data [22]. Despite promising results indicated by moderate to high area under the Receiver Operating Characteristic Curve (AUC-ROC) [24] scores (i.e., 0.6–0.9), most existing studies generally place less focus on designing modelling methodologies that explicitly account for small cohort sizes. Additionally, they do not discuss the reliability associated with classification decisions, which would reflect the risk, i.e., predicted probability, of a sample being diagnosed with a particular disease given the information, i.e., empirical probability, from the rest of the available samples. The omission of the latter, which is called *probability calibration* [25], is not just an occurrence in glycomic-based predictive modelling studies but is pervasive to many clinically-relevant machine learning (ML) applications [26]. Furthermore, there is value in using such models to infer insights into which features (i.e., relative abundance of glycan structures) drive model decisions using global model-agnostic interpretability techniques [27]. This additional information complements univariate statistical tests with methods that account for glycan interactions.

This work aims to address the above challenges by developing a comprehensive framework based on data analysis and ML that could enable the extraction of the biomarker potential of IgG N-glycans for disease diagnosis and monitoring. This work contributes to ongoing research efforts in the field by incorporating a series of ML algorithmic approaches used for tabular data into the relatively immature field of glycomic-based predictive modelling. Specifically, it integrates key concepts, such as the use of all available glycomic data for model training, optimisation and performance evaluation using nested cross-validation (NCV), places particular focus on probability calibration to ensure that the predicted probabilities calculated by the trained ML pipelines align with the empirical probabilities and uses global model-agnostic interpretability techniques to inspect how these well-calibrated probabilities are affected by changes in certain N-glycans. To the best of our knowledge, this is the first time that such a framework based on probability calibration has been considered in the field of glycomic-based predictive modelling studies. To build and test it, a published IgG N-glycomic dataset for colorectal cancer (CRC) was used [28], [29], which was large enough to facilitate the implementation of ML and represented a highly incident cancer type, screening and diagnosis of which would benefit from the development of minimally-invasive, sensitive, and low-cost tools [30], [31].

## 2 Materials and methods

**Fig. 1:**
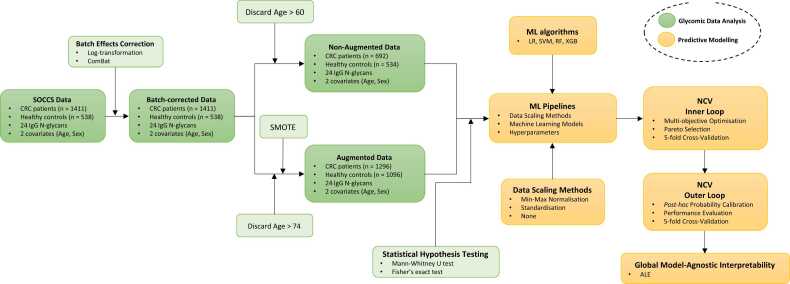
Schematic of proposed methodological framework for the construction, optimisation, and evaluation of ML pipelines using IgG N-glycomic data for disease risk stratification.

The proposed computational framework comprises two interconnected modules, namely the glycomic data analysis module and the predictive modelling module. A schematic of the proposed computational framework is presented in Fig. 1. All analyses were performed using Python 3.9.

### 2.1 IgG N-glycomic dataset description

In this work, a dataset from the Study of Colorectal Cancer in Scotland (SOCCS) cohort (1999–2006) was used [28], [29]. This includes the total area normalised relative abundance of 24 total IgG N-glycan peaks (hereafter referred to as *GPs*) derived from human plasma and measured by high-throughput ultra-performance liquid chromatography (UPLC). The specific structures corresponding to the GPs considered in this study expressed using the Oxford nomenclature and accompanied by their GlyTouCan accession numbers [32] are available in the Table S1. Furthermore, the SOCCS dataset includes anonymised sample identification (ID), analytical measurement information (sample date and plate used), demographic information about individuals in the cohort (sex, and age), and CRC status (control or cancer). The SOCCS dataset contained 1411 patients with pathologically confirmed CRC and 538 healthy controls. The dataset presented a notable imbalance in the number of CRC patients and healthy controls over the age of 60. The scarcity of older-aged healthy controls was overcome via two approaches: dropping all samples over 60 years old from the SOCCS dataset leading to the age, and sex-matched Non-Augmented (N-AUG) dataset and augmenting the SOCCS dataset to create the age, and sex-matched Augmented (AUG) dataset. The data augmentation strategy followed is presented in subsection 2.2.2.

### 2.2 Glycomic data analysis

This subsection concerns the data preprocessing steps that were carried out to prepare the glycomic data for predictive modelling tasks. Unless otherwise stated, data analysis was performed using the *numpy* [33] and *pandas* [34] packages.

#### 2.2.1 Batch effect correction

The presence of batch effects, characterised as undesirable technical variation, presents a significant challenge in the analysis of omics data, including glycomic data. Stemming from variations in sample processing, experimental conditions, or measurement technologies across different batches of samples, these effects can confound the true biological signal and lead to potential misinterpretation of the data and the inference of spurious associations [35]. Therefore, correcting for batch effects is crucial to ensure the validity and reproducibility of omics studies and to improve the overall robustness of downstream statistical analysis. In this study, batch effects included the year the samples were analysed and the plate used for the analytical measurements. Batch effects were corrected using the popular ComBat algorithm [36] using the *scanpy* [37] package after the GPs were first log-transformed [28], [38]. The ComBat algorithm was originally designed for batch effect correction in gene expression data and uses linear models and an empirical Bayes (EB) framework to correct for undesired technical variation [36].

#### 2.2.2 Data augmentation and statistical hypothesis testing

The dataset lacks healthy controls older than 60 years. Early epidemiological studies in the field have shown that the relative abundance levels of most IgG N-glycans are strongly associated with demographic variables of age and sex [39], [40], [41], thus confounding the analysis. To mitigate spurious associations between the GPs and CRC, this imbalance should be addressed prior to analysis and modelling. The first approach to address this imbalance was to exclude samples over 60 years old from the batch-corrected SOCCS dataset to yield the N-AUG dataset. However, to avoid discarding a large proportion of the available data (i.e., more than 700 samples), it was decided to also perform data augmentation using the Synthetic Minority Over-Sampling Technique (SMOTE) [42] implemented in the *imbalanced-learn* [43] package to generate the AUG dataset.

SMOTE works by first randomly selecting a sample, *a*, that belongs to the minority class (healthy controls in this study) and then finding the *k*-nearest neighbours (KNN) to *a* (*k = 3* in this study). One of the identified KNN samples, *b*, is randomly chosen to generate a synthetic sample as a linear combination of the values of *a* and *b* [42]. The SMOTE-based data augmentation strategy followed in this work incorporated knowledge of the distributions of the age and sex features across the control and CRC samples to ensure that subsequent classification decisions would be attributable to differences in the GPs and not artificial differences in these covariates as a result of data augmentation. Since no control samples over the age of 74 were available in the SOCCS dataset, this was considered the upper bound with respect to age and all older samples from both classes were dropped. Then, for the age range of 61 to 71, the data was oversampled to match the exact number of controls and CRC patients for each age. Finally, for the age range of 72–74, the data was oversampled so that the number of controls for each age would equal half the number of CRC patients. Throughout this process, the sex variable has been one-hot encoded and the proportion of males to females in the synthetic minority samples was kept approximately constant by random assignment.

Statistical hypothesis testing was performed before and after the described data augmentation strategy to assess whether differences in the underlying distributions of the covariates were statistically significant. The null hypothesis for each test was that there were no statistically significant differences in the age and sex distributions between the two classes at a 95% confidence level. The Mann-Whitney U test was used for the age and GP variables, and Fisher’s exact test was used for the sex variable, both implemented using the *scipy* [44] package. In all statistical analyses presented in this study, the false discovery rate in multiple testing was controlled via the Benjamini-Hochberg (BH) procedure to produce the q-values (i.e., adjusted p-values) [45].

### 2.3 Predictive modelling

This subsection describes the different steps that were taken to build, optimise, and evaluate ML pipelines for binary classification tasks corresponding to CRC screening and diagnosis. Unless otherwise stated, the *scikit-learn* [46] package was used throughout the subsequent analysis.

#### 2.3.1 Selection of ML algorithms

In this work, four ML classification algorithms were considered, namely logistic regression (LR), support vector machines (SVM), random forest (RF), and XGBoost (XGB).

LR describes the relationship between features and outcomes by incorporating it into the exponential of a logistic function. This adjustment allows the output to fall between 0 and 1, effectively converting a regression problem into a probabilistic classification framework [47].

SVM focuses on the geometry of the data and operates by finding an optimal hyperplane that effectively separates the different classes present in the feature space. The central premise of SVM is the idea of maximising the margin around the separating hyperplane, creating the largest possible distance between the decision boundary and any sample in the training dataset to enhance the generalisation ability of the model [48]. In cases where classes are not linearly separable in the original feature space, SVM cleverly projects the data into a higher-dimensional space via a kernel function, where the data then becomes linearly separable. This transformation ability, termed the *kernel trick*, gives SVM the flexibility to handle complex, non-linear relationships [49].

RF, a bagging-based ensemble algorithm, integrates a multitude of decision tree classifiers, each casting an individual vote based on a randomly selected subset of features and the classification of a new sample is subsequently conducted based on majority voting [50], [51]. This approach effectively harnesses the power of collective decision-making, improving the robustness of the model by mitigating the risk of overfitting often associated with single decision trees [52].

XGB, similarly to Random Forest, also derives from decision-tree-based ensemble learning. However, it adopts a distinct sequential learning strategy inspired by the gradient boosting framework [53]. As opposed to the parallel tree-building process in RF, XGB iteratively adds new trees to the ensemble, where each new tree is built to correct the errors made by the existing ensemble. An embedded gradient descent algorithm minimises these errors, thus improving model performance with this iterative refinement, lending XGB enhanced predictive power and robustness [54].

These four algorithms were chosen as a means of incorporating a diverse range of inductive biases into the classification tasks under investigation. Furthermore, RF and SVM have been widely used in ML-based disease diagnosis tasks in the literature [55], [56], and XGB is considered one of the most effective algorithms for classification tasks based on tabular data [57] having garnered a lot of attention due to its success in recent ML challenges [58]. Regarding glycomic-based predictive modelling studies, LR, SVM and RF have been employed in the past [22], [59], [60], [61], however, to the best of our knowledge, this is the first time that a predictive modelling study using glycomic data incorporates XGB in its analysis. XGB was implemented using the *xgboost* [54] package.

#### 2.3.2 Construction and optimisation of ML pipelines

Binary classification tasks were addressed using configurations of different types of algorithms and their hyperparameters, which represent different steps of the learning process, hereafter referred to as *ML pipelines*. In particular, a ML pipeline is characterised by the following components: *(i)* a data scaling algorithm, which can be used to normalise (i.e., use the minimum and maximum values for scaling) and/or standardise (i.e., transform data to have zero mean and unit standard deviation) continuous features, including the GPs, *(ii)* a ML algorithm, which can be any of the four (i.e., LR, SVM, RF, XGB) that were considered in this study, *(iii)* a set of hyperparameters for the selected ML algorithm, which depends on the type of algorithm chosen and is determined given a hyperparameter grid (Table S2).

**Fig. 2:**
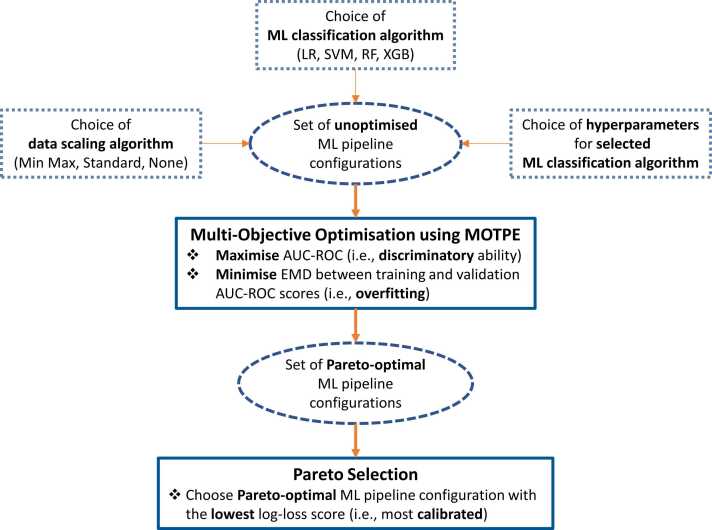
Schematic of proposed methodology for the construction and optimisation of ML pipelines using the N-AUG and AUG datasets. This procedure was implemented for fifty multi-objective optimisation trials per fold of the inner loop of the NCV scheme. The selected Pareto-optimal configuration was subsequently calibrated and evaluated on the folds of the outer loop of the NCV scheme. LR: logistic regression; SVM: support vector machine; RF: random forest; XGB: XGBoost; Min Max: min-max normalisation; Standard: standardisation; None: No data scaling; AUC-ROC: area under the Receiver Operating Characteristic Curve; EMD: Earth Mover’s Distance.

The selection of the constituent components and the optimisation of each pipeline was carried out via a multi-stage methodology schematically shown in Fig. 2. Given the N-AUG and AUG datasets, an initial pipeline is configured using random sampling, and then multi-objective optimisation is carried out to simultaneously maximise the discriminatory ability of the pipeline, which is quantified by the AUC-ROC score and minimise overfitting, which is quantified as the first Wasserstein distance, also known as *Earth Mover’s Distance* (EMD), between the training and validation AUC-ROC scores. The choice of these particular objectives was made to encourage the production of non-overfitted pipelines with high classification performance.

Multi-objective optimisation was carried out using the multi-objective Tree-structured Parzen Estimator (MOTPE) algorithm [62]. MOTPE is an extension of the Tree-structured Parzen Estimator (TPE), a type of sequential model-based, probabilistic optimisation strategy that iteratively refines the search for the optimal pipeline configuration based on the results of previous optimisation trials [63]. Similarly to single-objective TPE, MOTPE generates two Gaussian Mixture Models (GMMs), *l(x)* and *g(x)*, each corresponding to a high-performing and low-performing region of the search space (i.e., choice of data scaling algorithm, ML algorithm, and set of hyperparameters for the selected ML algorithm). While single-objective TPE would preferentially sample new solutions (i.e., optimal sets of data scaling algorithm, ML algorithm, and hyperparameters for selected ML algorithm) by maximising the ratio between *l(x)* and *g(x)* [64], in a multi-objective context the distinction between high and low performance is not binary and straightforward, since superior performance with respect to one objective does not also guarantee superior performance with respect to the other objectives, as these may be conflicting [65]. To address this, MOTPE utilises a Pareto dominance-based strategy to determine the compartmentalisation of the search space and produce Pareto-optimal solutions in subsequent optimisation trials. The implementation of MOTPE was considered in this study as it is well-suited for multi-objective optimisation involving discrete and continuous decision variables due to its probabilistic approach, effectively managing mixed variable types [66] and ensures that subsequent solutions are based on knowledge from previous trials, which is not the case with other popular hyperparameter tuning approaches, such as grid search and random sampling.

Given that the output of MOTPE is a set of Pareto-optimal solutions, which represent optimally performing non-overfitted pipelines, a further consideration needs to be made regarding the selection of a single solution that would be considered the “best” one to be used for the final performance evaluation. By definition, Pareto-optimal solutions form a set of mathematically commensurable solutions [67], and an additional selection strategy needs to be adopted to determine which of the solutions is the most desirable. In this work, the selected Pareto-optimal ML pipeline is the one that scores the lowest with respect to logarithmic loss, also most commonly referred to as *log-loss*. Log-loss is a popular performance metric in classification problems that provided a measure of error heavily influenced by the confidence of the prediction. It quantifies how close the predicted probabilities are to the corresponding true values and penalises confident, incorrect predictions (i.e., high predicted probability to the wrong class) [68]. Thus, it is a metric that can be used to choose a non-overfitted, high-performing ML pipeline that is also reliable (i.e., well-calibrated), since log-loss inherently encourages the adjustment of predicted probabilities to align as closely as possible with empirical probabilities [25], [68]. As described in subsection 2.3.3, probability calibration usually takes place as a *post-hoc* procedure, meaning that it is carried out once the underlying model or pipeline has already been trained and tuned with a use of an additional model referred to as *calibrator* [25]. However, it has been suggested to embed considerations about probability calibration into the learning process itself to achieve more reliable and robust results [25], which is particularly important in clinical applications such as the one presented herein, where careful and informed decision-making is required. The MOTPE optimisation and Pareto selection procedure were performed using the *optuna* [69] package.

#### 2.3.3 *Post-hoc* probability calibration and performance evaluation of ML pipelines

The aim of *post-hoc* probability calibration is to leverage a hold-out subset of the available data, termed the *calibration set*, to generate a mapping for a pre-trained ML model to refine its predicted probabilities to reflect observed true probabilities when tested on new, unseen data [25]. Among the different calibration techniques available for binary classifiers, including logistic calibration, also known as *Platt scaling* [70], isotonic regression, also referred to as *ROC convex hull* method [71], and beta calibration [72], this study used the inductive Venn-Abers predictor (IVAP) approach for probability calibration [73]. IVAP is a regularised calibration method, which is a special case of well-calibrated Venn-Abers predictors and is based on isotonic regression [73], [74]. Once fitted to the calibration set, IVAP generates a probability prediction interval for each test sample by performing two separate fits of isotonic regression. This interval, denoted as $\left[p_{0},p_{1}\right]$, is characterised by a lower probability bound $p_{0}$ and an upper probability bound $p_{1}$, which represent the well-calibrated confidence levels for a particular class of a test sample, thus quantifying the uncertainty associated with the calibrated predicted probability that a sample would belong into a particular class, usually the positive one (i.e., here CRC patients) [74]. To obtain a single calibrated predicted probability for each class of a sample, the minimax approach can be used to obtain the score for the positive class and the negative class as follows [73], [74]: $p_{\mathrm{pos}}=p_{1}/(1-p_{0}+p_{1})$ and $p_{\mathrm{neg}}=1-p_{\mathrm{pos}}$. The IVAP technique overcomes the known reported limitation of the standard isotonic regression approach, which is prone to overfitting when presented with smaller calibration sets, and unlike Platt scaling and beta calibration, which are both parametric calibration methods, IVAP does not make specific assumptions about the form of the underlying distribution of the class labels [73]. Thus, IVAP was selected as the *post-hoc* calibration method used in this study, as it seamlessly aligns the overarching aim of this study to design a flexible and generalisable methodology to build and reliably evaluate ML pipelines using glycomic data for disease risk stratification. The Python implementation of IVAP used in this study can be found here [75]. *Post-hoc* calibration performance was assessed using the expected calibration error (ECE) and log-loss [76], [77] metrics.

Holding out a subset of data just for testing reduces the amount of data available for training, which can worsen model performance [78]. Especially in the case of glycomic datasets, which tend to be small [22], a loss of samples to performance evaluation is not favourable. To that end, NCV offers an approach to avoid this pitfall, where, instead of employing a strict, one-time partitioning of the data, it uses a scheme of multiple rounds of partitioning (i.e., folds) so that all folds serve as validation and test sets [79], [80]. This allows NCV to produce unbiased performance estimates as compared to standard cross-validation, where model selection and performance estimation are done on the same subsets of each iteration (i.e., validation sets), which has been shown to produce biased performance estimates [78], [81].

In this study, the NCV protocol was employed for the binary classification tasks using stratified 5-folds for the inner and the outer loop. The former was responsible for carrying out the multi-objective optimisation of the ML pipelines using 50 optimisation trials for each fold of the inner loop (see subsection 2.3.2). The outer loop was responsible for both *post-hoc* probability calibration and performance evaluation, where 60% of the data from each outer loop fold were used for the former and 40% for the latter. Performance was assessed using the AUC-ROC metric, with the sensitivity and specificity of the ML pipelines determined using a decision threshold of 0.5. Additionally, the count of misclassified samples versus the total sample count across all test folds of the outer NCV loop for different age ranges was monitored. These counts were computing with the same decision threshold of 0.5.

#### 2.3.4 GP effects using global model-agnostic interpretability

In the context of clinical decision-making, it would also be valuable not only to infer if an individual has a particular chronic disease given a set of GPs, but also to determine how the risk of getting diagnosed with this particular chronic disease is expected to vary given changes in these GPs. To achieve this, trained ML pipelines for disease classification can be inspected using global model-agnostic interpretability techniques. Global techniques allow the investigation of how feature effects impact the model’s output on average, across all samples, rather than focusing on understanding individual predictions (i.e., local interpretability). In parallel, model-agnostic techniques offer a way to interpret these predictions irrespective of the model’s internal structure or complexity [27]. To allow the investigation of the effects of specific GPs on CRC risk, while also accounting for their interactions, the Accumulated Local Effects (ALE) technique was implemented in this study [82]. ALE isolates the effects of individual features by examining their impact in small intervals of their range, thus allowing one to attribute any changes in the model predictions to these local changes in the feature of interest. The term *accumulated* stems from the idea that these local effects are averaged across the entire range of a feature, giving a global interpretation of the feature’s effect on the model outputs [82]. Unlike other global model-agnostic interpretability techniques, such as Partial Dependence Plots (PDP) [83] and Permutation Feature Importance (PFI) [84], ALE is more effective when dealing with correlated features due to its local approach, thus allowing a clearer view of individual feature effects [27]. In this study, the ALE technique was implemented using the *alibi* [85] package.

## 3 Results

### 3.1 CRC screening using the N-AUG dataset

**Fig. 3:**
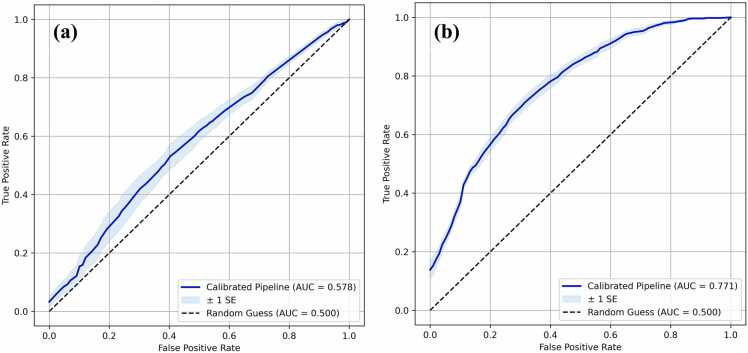
ROC for the ML pipelines evaluated via NCV using the (a) N-AUG and (b) AUG datasets. AUC denotes the mean area under the curve for all five test folds of the outer loop and SE denotes the standard error of the mean.

Due to the small number of healthy control samples over the age of 60 years in the original SOCCS dataset, the first approach was to discard all samples from patients over 60 from both classes, thus creating the N-AUG dataset (see subsection 2.2.2). By implementing the workflow presented in Fig. 1, the mean AUC-ROC test score achieved was 0.578 (Fig. 3a), with the corresponding 95% confidence interval as evaluated by the NCV approach being (0.524, 0.631). Considering that the 95% confidence interval of the mean did not include an AUC-ROC score of 0.5, which corresponds to a random guess, this result indicates that the N-AUG dataset contains predictive information for CRC classification. However, the discriminatory ability of the underlying ML pipelines is not high enough to be acceptable for screening applications. This is further supported by the low mean sensitivity and specificity values of 0.708 and 0.380, respectively. The former indicates that, on average, the underlying ML pipelines across all outer folds of NCV have difficulty determining true positives, which leads to a high mean false negative rate (i.e., 1-sensitivity: 0.292). This means that certain CRC patients are expected to be misclassified as healthy. Looking at the count of misclassified samples within different age ranges across all test folds of the outer NCV loop (Fig S1), it was discerned that the underlying ML pipelines struggled to correctly classify the samples equally, irrespectively of the total sample count in each age range. This suggests that there is no clear pattern of GP changes within these age groups that could be exploited to differentiate between CRC patients and healthy controls. In terms of the ML pipelines selected, LR was chosen as the underlying classification algorithm by 3 out of the 5 folds of the inner NCV loop, with SVM and RF chosen once each (Table 1). Note that the classifier selection was conducted by choosing the Pareto-optimal solution generated by a particular fold that scored the lowest with respect to log-loss to encourage the selection of the best calibrated ML pipeline relative to the rest of the Pareto-optimal solutions (see subsection 2.3.2). The different choices made by NCV across the inner NCV folds indicate high variance, which could be attributed to the fact that the N-AUG dataset contains a relatively small number of samples (i.e., 1226).

**Table 1:** Configuration of machine learning pipelines for all folds of the inner NCV loop using the N-AUG and AUG datasets grouped by classifier type. Min Max: min-max normalisation; Standard: standardisation; None: No data scaling; C: regularisation parameter; L1: Lasso penalty term; L2: Ridge penalty term; Elastic Net: Combination of L1 and L2; Criterion: tree-specific splitting criterion; GBTree: tree-based booster; DART: dropout regularized tree-based gradient booster.

**Algorithm**	**Dataset**	**Hyperparameters**				
		**Scaler**	**C**	**Penalty**	**Solver**	**L1 ratio**
**LR**	N-AUG N-AUG N-AUG	None None None	0.19 0.19 0.36	L1 L1 Elastic Net	SAGA SAGA SAGA	- - 0.85
		**Scaler**	**C**	**Kernel**		
**SVM**	N-AUG	None	0.001	Polynomial		
		**Scaler**	**Estimators**	**Max Depth**	**Criterion**	
**RF**	N-AUG	None	40	2	Entropy	
		**Scaler**	**Estimators**	**Max Depth**	**Booster**	**Gamma**
**XGB**	AUG	None	400	6	GBTree	8.44
	AUG	Standard	360	5	DART	8.93
	AUG	Standard	320	5	DART	8.19
	AUG	Min Max	240	6	DART	11.18
	AUG	Min Max	240	6	DART	11.18

### 3.2 CRC screening using the AUG dataset

As discussed in subsection 2.2.2, data augmentation using SMOTE was carried out to address covariate imbalance in the dataset and allow the use of all existing plasma samples. The quality of the employed data augmentation strategy was assessed using statistical hypothesis testing. Specifically, the statistical difference between the covariates (i.e., age and sex) across the control and CRC classes remained not significant at a confidence level of 95% before (q=0.17) and after (q=0.08) augmentation, which indicated that subsequent classification decisions based on the AUG dataset could be attributed to observed differences in the GPs and not to differences in these covariates as a result of data augmentation. It should be noted, however, that SMOTE can negatively impact the calibration performance of classification algorithms [86], which is particularly undesirable in clinical applications. This consideration further supports the use of post-hoc calibration techniques, such as IVAP, in this study.

Looking at the results for the AUG dataset, the mean AUC-ROC test score is significantly improved compared to the N-AUG dataset, leading to a performance of 0.771 (Fig. 3b), with the corresponding 95% confidence interval being (0.746, 0.795). The resulting performance estimate confirms the hypothesis that the originally discarded samples contained valuable information regarding CRC classification and improves on similar estimates that were generated using IgG N-glycomic information from the SOCCS dataset in previous studies, namely a mean AUC-ROC of 0.755 in [28] and a mean AUC-ROC of 0.660 in [29]. Additionally, both the mean sensitivity and specificity, 0.819 and 0.557, respectively, increased as compared to the N-AUG dataset, which leads to a pronounced decrease of the mean false negative rate, now equal to 0.181. This finding suggests that the underlying ML pipelines are, on average, better able to detect true positives. However, it should be stressed that the moderate mean specificity achieved by these ML pipelines indicates that this high true positive rate will be accompanied by a relatively high false positive rate, thus being incumbent upon the user to consider the relative cost of false negatives and false positives in different clinical settings (see Section 4).

**Fig. 4:**
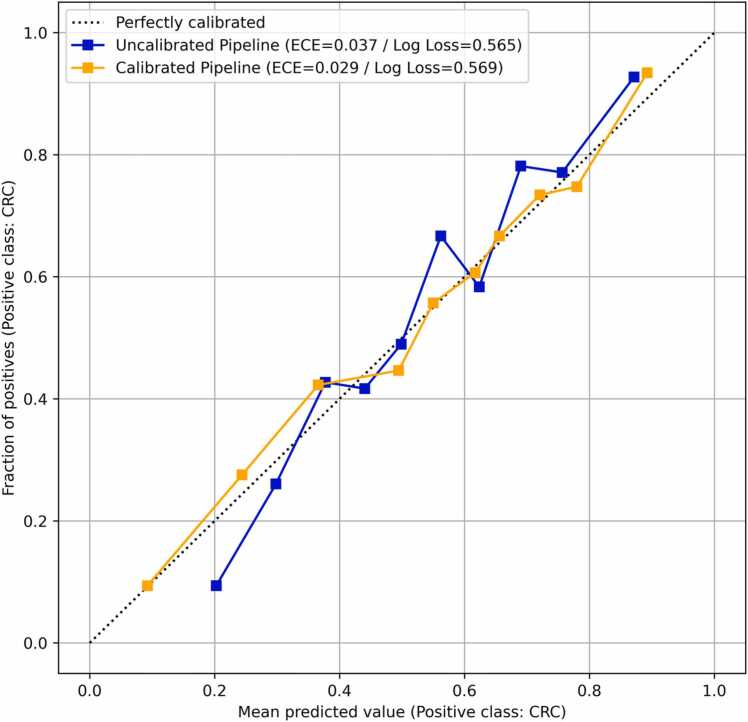
Reliability diagram constructed using the predicted probabilities obtained by the ML pipelines evaluated on the five test folds of the NCV scheme using the AUG dataset. Probability calibration was carried out using IVAP and the quality of calibration was assessed using ECE and log-loss.

Looking at the count of misclassified samples within different age ranges (Fig. S2), the underlying ML pipelines exploit the GP changes in older samples (i.e., 59–63, 64–68, 69–73) for correct classifications. This trend is consistent with the fact that CRC is more prevalent in people over 50 years old in Scotland and the United Kingdom [87], [88]. It should be highlighted that the oversampling of the controls in this age range was based on a small number of samples and the results should be validated in independent cohorts. Furthermore, the selection of pipeline constituent components was robust, leading to the selection of the XGB model in all five NCV inner loops (Table 1). Fig. 4 presents the resulting reliability diagram, which was constructed based on all five folds of the NCV outer loop. The implementation of IVAP improves calibration both qualitatively and quantitatively, thus ensuring that the produced predicted probabilities closely align with the observed empirical probabilities in the dataset. This enables the ML pipelines to produce reliable estimates of the risk associated with classifying each sample as cancerous or not. Overall, these results suggest that human plasma IgG N-glycans may aid in CRC screening as sole biomarkers or in combination with other CRC-specific biomarkers in an integrated diagnostic workflow [22], [29].

### 3.3 GP effects based on the AUG dataset

**Fig. 5:**
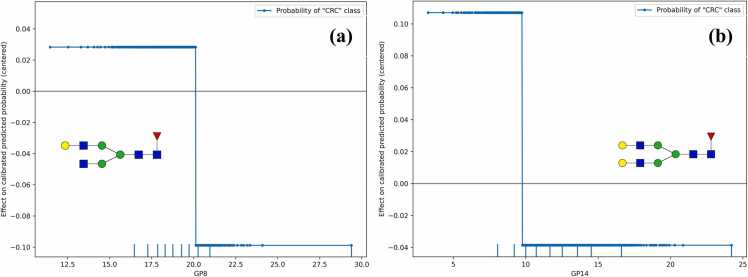
ALE plots for the core-fucosylated galactosylated IgG N-glycans (a) GP8 (FA2[6]G1) and (b) GP14 (FA2G2).

Using ALE, it is possible to gain insight into the effect of specific GPs on the calibrated predicted probabilities of CRC classification (i.e., risk of CRC screening and diagnosis). To achieve this, a final, optimised, and calibrated ML pipeline produced on the AUG dataset was required (henceforth referred to as *final AUG ML pipeline*), as NCV does not return a final ML pipeline to be promptly deployed, but only acts as a comprehensive evaluation framework (see Section 2.3.3). To this end, standard 5-fold cross-validation was first performed on the 90% of the AUG dataset to obtain the ML pipeline configuration with the best hyperparameters, then the resulting pipeline was re-trained on same 90% of the dataset and was finally calibrated using IVAP on the remainder 10%. The final AUG ML pipeline led to the selection of the XGB classification algorithm as the underlying classifier, which is in accordance with the results obtained by the NCV scheme (Section 3.2). Using this pipeline, the ALE values with respect to the well-calibrated probability of the CRC class for all features were computed. Interestingly, the ALE values for the age and sex variables were both zero, which suggests that both covariates were properly balanced across the two classes as the pipeline did not leverage changes in these variables during training to infer CRC risk. Out of the 24 GPs, 8 have non-zero ALE values, namely GP1, GP4, GP8, GP11, GP14, GP15, GP16, and GP22, which suggests that these changes in these GPs were the main drivers behind decisions about CRC status. Most of these GPs (i.e., 6 out of 8) are core-fucosylated neutral N-glycans. This observation is in agreement with previous findings using the SOCCS dataset [28], [29], where it was shown that changes in core-fucosylated neutral N-glycans are involved in CRC. Indicatively, Fig. 5 depicts the ALE plots for two of these GPs, which are core-fucosylated galactosylated N-glycans, namely GP8 (FA2[6]G1) and GP14 (FA2G2).

## 4 Discussion

Bioinformatics efforts are underway to incorporate glycosylation into the central dogma of biology due to its ubiquitous presence in many aspects of human physiology and pathophysiology [89], [90]. Moreover, in recent years, glycan data analysis using open-source tools has emerged. Notably the development of resources such as *Glycowork* [91] provides a vast repertoire of computational tools to help process and analyse glycan data. Further attempts to design customisable methodologies to interrogate glycomic data could aid the quantitative analysis of disease-related glycan alterations in human plasma proteins. The present study uses human plasma-derived GPs from the large SOCCS cohort to assess whether they could be employed to screen CRC patients. This was achieved via a series of binary classification tasks, which were addressed using a modular methodology designed to balance the trade-offs between practical constraints, such as available computational resources, and level of detail in the results, such as NCV for the performance evaluation of the resulting ML pipelines.

This work adds to existing CRC-related glycomic studies [28], [29], [92], [93], [94], [95] by yielding promising results with respect to the observed AUC-ROC scores. Particularly in the AUG scenario, the AUC-ROC score was on par with the ones obtained in the referenced glycomic studies. Finally, the results of the global model-agnostic interpretability approach in this study pointed to neutral fucosylated N-glycans as potential biomarkers of CRC, recapitulating observations found in the literature using different statistical approaches, such as multiple regression analysis [28]. Interestingly, model inspection came to this conclusion without knowing the structural characteristics of the underlying N-glycans, but solely via the exploitation of the patterns formed by their GPs. The proposed ML pipelines now need to be independently validated against additional CRC cohorts before implementing them in a clinical setting given that more evidence about the role of plasma-derived N-glycans in CRC is required. Future extensions of this framework will attempt to embed structural information about the N-glycan biosynthetic network itself into predictive tasks to assess whether this type of information can help improve the insights obtained by trained and optimised ML pipelines for CRC and other diseases.

A key concept in the proposed computational framework was the ability to output calibrated predicted probabilities for the patient class based on which the risk of being classified with that particular disease is quantified. We believe that this is an essential aspect in facilitating the adoption of glycan-based biomarkers in clinical settings. Probability calibration ensures that the predicted probabilities for the disease class provide a reliable risk stratification for the patients. For example, popular metrics for performance evaluation in classification, including sensitivity and specificity, require the determination of a decision threshold to place all samples in their respective classes to be then compared against their true labels. If the predicted probabilities are miscalibrated, then the resulting class membership would be invalid and the reported performance metrics likely misleading. Additionally, well-calibrated probabilities can be used reliably to determine the decision threshold in a cost-sensitive manner. When it comes to screening applications, false negatives can be more detrimental than false positives, since patients would be wrongly considered as healthy, which would hinder timely clinical interventions. In such cases, the decision threshold can be lowered accordingly to only make negative predictions if sufficiently certain. Our framework allows the user to specify what they deem as appropriate relative costs between the false negatives and false positives, which is an application-dependent decision based on the clinical context. It should be noted that the use of poorly calibrated predicted probabilities would invalidate such an analysis.

Using global model-agnostic interpretability methods on trained ML pipelines to infer insights into which GPs influence classification decisions can potentially lead to a more dynamic, patient-centred approach, which could help the early identification of disease onset or progression, possibly even before clinical symptoms appear. The management of chronic diseases, such as autoimmune disorders and cancer, could benefit from such an approach, to hopefully provide an incremental improvement in patient outcomes. Furthermore, gaining insight into how the risk of diagnosis is affected by changes in GPs could serve to customise treatment strategies by monitoring patient response to treatment [22]. Finally, understanding how GP levels impact the associated diagnostic risk could also help clinicians to better articulate the patient’s condition and the reasoning behind their treatment plan, thereby fostering increased patient understanding, engagement, and trust in recommended therapeutic interventions. It should be noted that the implementation of appropriate global model-agnostic interpretability approaches, such as ALE, should be based on ML models and/or pipelines that have been carefully constructed, optimised, and evaluated to avoid overfitting and that can produce calibrated probabilities as reliable indicators of diagnostic risk.

## 5 Conclusions

This study proposed a comprehensive ML-based framework to analyse N-glycomic data for disease risk stratification. This framework was built and tested using a published CRC dataset producing promising results for the use of IgG glycan-based predictive tools for stratifying CRC risk that could help prioritise those with non-specific symptoms for early access to diagnostic modalities. The present framework is modular and can be implemented for the investigation of different disease types. Considering the already delineated potential of IgG N-glycans as disease biomarkers, particularly characterised by the impressive plasticity to various pathophysiological stimuli, it is emphasised that computational tools such as the one presented in this study that account for the intricacies involved in clinical decision-making could act as a useful proponent in expediting the adoption of glycan-based biomarkers in clinical settings.

## CRediT authorship contribution statement

**Konstantinos Flevaris (KF)**: Conceptualization, Data curation, Formal Analysis, Methodology, Software, Visualization, Writing – original draft. **Joseph Davies (JD)**: Conceptualization, Formal Analysis, Methodology, Software, Visualization, Writing – original draft. **Shoh Nakai (SN)**: Conceptualization, Formal Analysis, Investigation, Methodology, Software, Visualization Writing – original draft. **Frano Vučković (FV)**: Investigation, Writing – review & editing. **Gordan Lauc (GL)**: Investigation, Writing – review & editing. **Malcolm G Dunlop (MGD)**: Investigation, Writing – review & editing. **Cleo Kontoravdi (CK)**: Conceptualization, Methodology, Resources, Supervision, Writing – review & editing.

## Declaration of Competing Interest

Gordan Lauc is the founder and CEO of Genos Ltd. Frano Vučković is employee of Genos Ltd. The remaining authors declare that the research was conducted in the absence of any commercial or financial relationships that could be construed as a potential conflict of interest.

## Data Availability

The source code necessary for reproducing the results presented in this paper is openly available on GitHub at https://github.com/kf120/IgG_Nglycan_CRC_classification_paper.
