# Supplementary material for: Machine learning framework to extract the biomarker potential of plasma IgG N-glycans towards disease risk stratification
Source: Comput Struct Biotechnol J. 2024 Mar 11;23:1234–43. doi: 10.1016/j.csbj.2024.03.008 (PMC10973724; doi:10.1016/j.csbj.2024.03.008)

**Supplementary Information**

**Machine learning framework to extract the biomarker potential of plasma IgG N-glycans towards disease risk stratification**

**Authors**

Konstantinos Flevaris^a, ┴, *^, Joseph Davies^a, ┴^, Shoh Nakai^a, ┴^, Frano Vuckovic^b^, Gordan Lauc^b, c^, Malcolm G. Dunlop^d^, Cleo Kontoravdi^a,^*

**Affiliations**

^a^ Department of Chemical Engineering, Imperial College London, London SW7 2AZ, United Kingdom

^b^ Genos Glycoscience Research Laboratory, Zagreb 10000, Croatia

^c^ Department of Biochemistry and Molecular Biology, Faculty of Pharmacy and Biochemistry, University of Zagreb, Zagreb, Croatia

^d^ Colon Cancer Genetics Group, Institute of Genetics and Cancer, Cancer Research UK Scotland Centre, University of Edinburgh and Medical Research Council Human Genetics Unit, Edinburgh, United Kingdom

Research Article submitted to: Computational and Structural Biotechnology Journal

________________________________________

┴ Authors to whom **co-first author** status is assigned.

* Authors to whom **correspondence** should be addressed.

E-mail address: [k.flevaris21@imperial.ac.uk](mailto:k.flevaris21@imperial.ac.uk)

E-mail address: [cleo.kontoravdi@imperial.ac.uk](mailto:cleo.kontoravdi98@imperial.ac.uk)

**Table S1**: Glycan structures corresponding to the glycan peaks (GP) considered in the present study. All glycans measured in this study correspond to total IgG glycans.

| GP | Oxford Nomenclature | Glycan Structure | GlyTouCan ID |
| --- | --- | --- | --- |
| GP1 | FA1 | 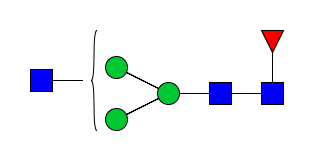 | G14576KZ / G69987TD |
| GP2 | A2 | 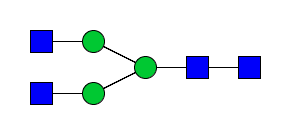 | G88876JQ |
| GP3 | A2B | 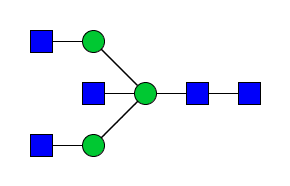 | G76241TN |
| GP4 | FA2 | 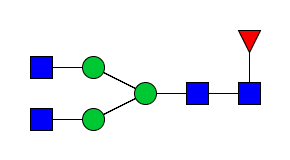 | G65984FE |
| GP5 | M5 | 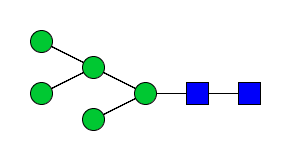 | G03652TR |
| GP6 | FA2B | 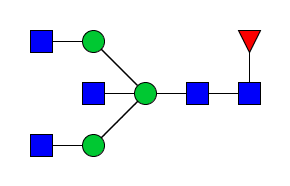 | G60956VN |
| GP7 | A2G1 | 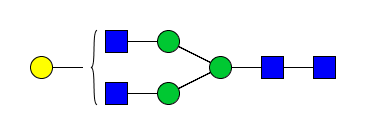 | G36859SD / G44754DF |
| GP8 | FA2[6]G1 | 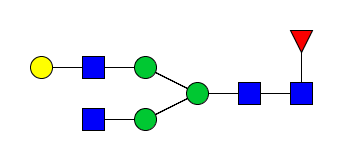 | G29024OJ |
| GP9 | FA2[3]G1 | 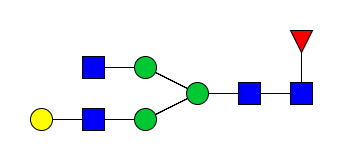 | G53582JE |
| GP10 | FA2[6]BG1 | 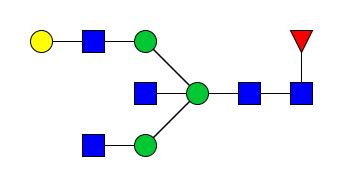 | G92575UZ |
| GP11 | FA2[3]BG1 | 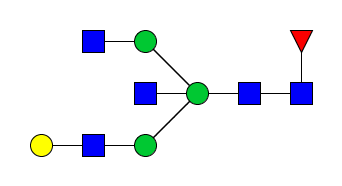 | G20079RG |
| GP12 | A2G2 | 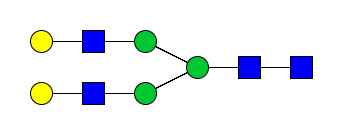 | G66741YQ |
| GP13 | A2BG2 | 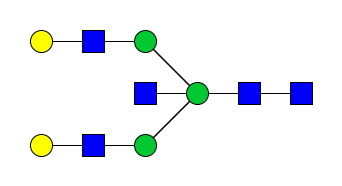 | G86522OD |
| GP14 | FA2G2 | 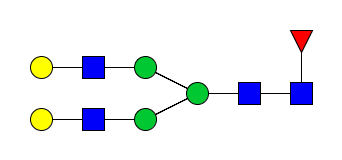 | G00998NI |
| GP15 | FA2BG2 | 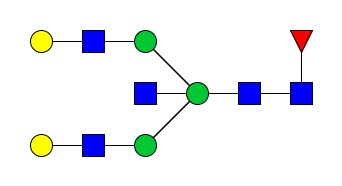 | G74325TG |
| GP16 | FA2G1S1 | 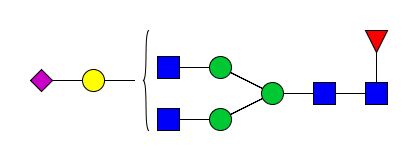 | G71782CJ / G19447ZX |
| GP17 | A2G2S1 | 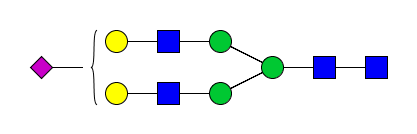 | G01670UQ / G37591JC |
| GP18 | FA2G2S1 | 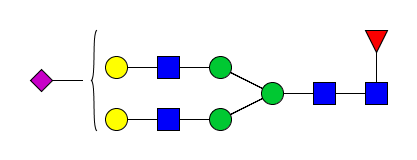 | G01361CV / G88242BL |
| GP19 | FA2BG2S1 | 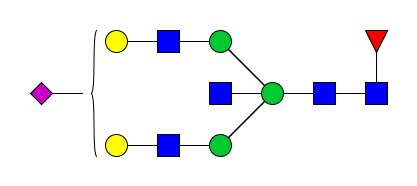 | G42295MA |
| GP20 | - | - | - |
| GP21 | A2G2S2 | 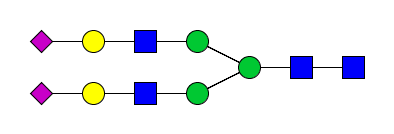 | G73866ZM |
| GP22 | A2BG2S2 | 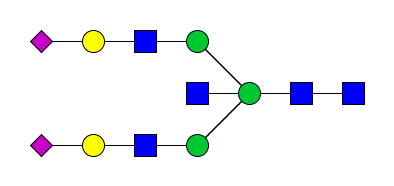 | G14854WL |
| GP23 | FA2G2S2 | 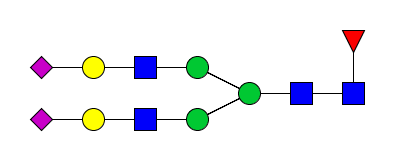 | G72954XC |
| GP24 | FA2BG2S2 | 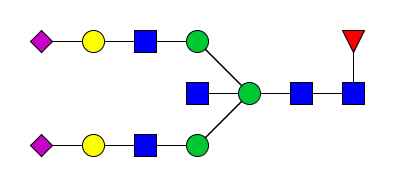 | G48713OY |

**Table S2**: Hyperparameter grid considered for the construction of the machine learning pipelines in this study.

LR: logistic regression; SVM: support vector machine; RF: random forest; XGB: XGBoost; L1: Lasso penalty term; L2: Ridge penalty term; Elastic Net: Combination of L1 and L2; RBF: radial basis function; Criterion: tree-specific splitting criterion; Log-loss: logarithmic loss; GBTree: tree-based gradient booster; GBlinear: linear gradient booster; DART: dropout regularized tree-based gradient booster

| Algorithm | Hyperparameter | Type | Range/Options | Step |
| --- | --- | --- | --- | --- |
| LR | Penalty | Categorical | L1, L2, Elastic Net | - |
|  | C | Float | 0.05 – 0.75 | - |
|  | Solver | Categorical | SAGA | - |
|  | L1 ratio | Float | 0 – 1 | - |
| SVM | Kernel | Categorical | Linear, Polynomial, RBF, Sigmoid | - |
|  | C | Float | 0.001 – 0.05 | - |
|  | Degree | Integer | 2 – 3 | 1 |
|  | Shrinking | Categorical | False, True | - |
| RF | Max depth | Integer | 2 – 4 | 1 |
|  | Estimators | Integer | 20 – 400 | 20 |
|  | Criterion | Categorical | Gini, Entropy, Log-loss | - |
| XGB | Max depth | Integer | 4 – 6 | 1 |
|  | Estimators | Integer | 20 – 400 | 20 |
|  | Booster | Categorical | GBTree, GBlinear, DART | - |
|  | Gamma | Float | 8 – 15 | - |

**Figure S1**: Misclassified and total counts by age range across all outer loop test folds of NCV using the N-AUG dataset


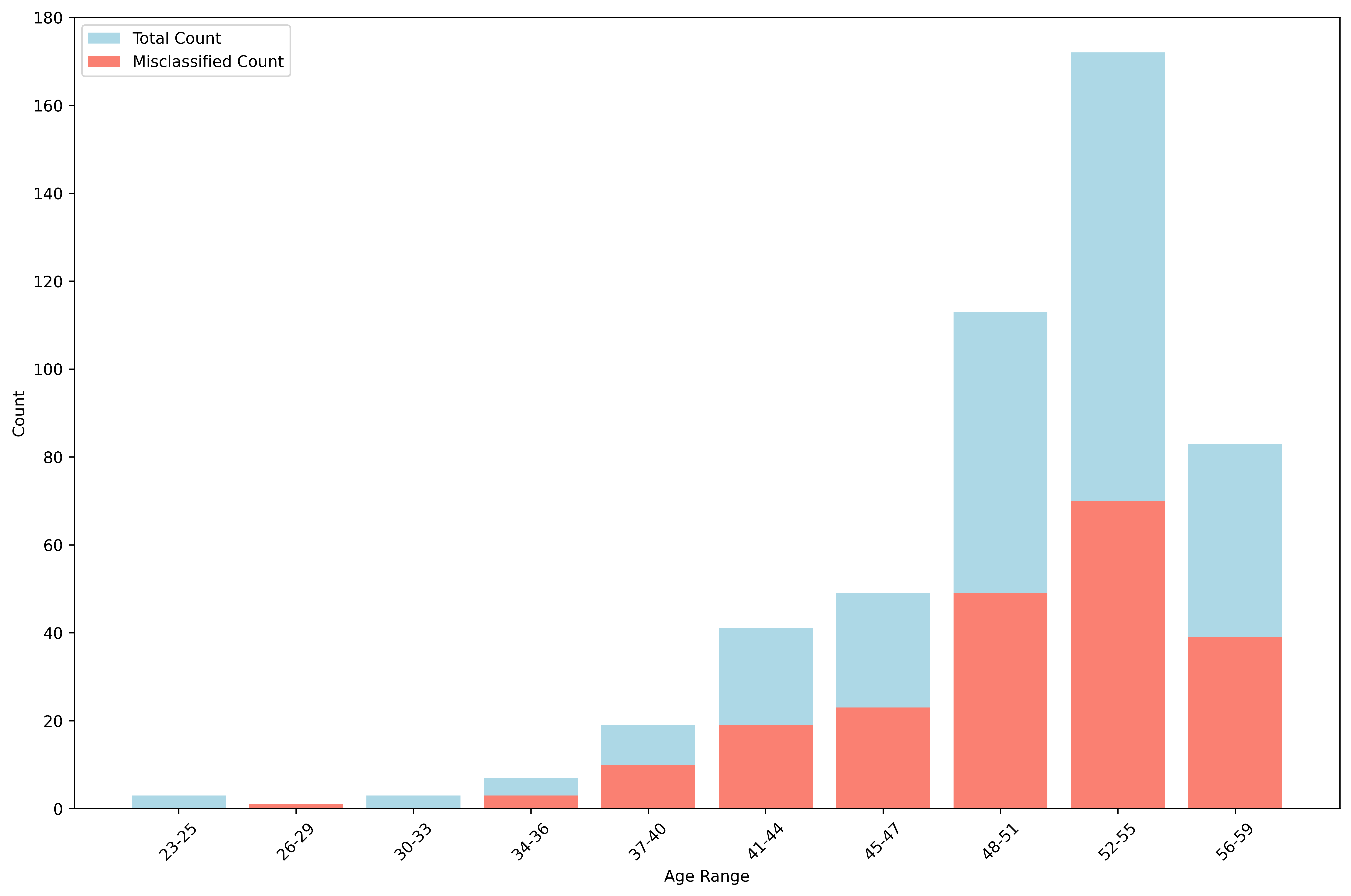


**Figure S2**: Misclassified and total counts by age range across all outer loop test folds of NCV using the AUG dataset


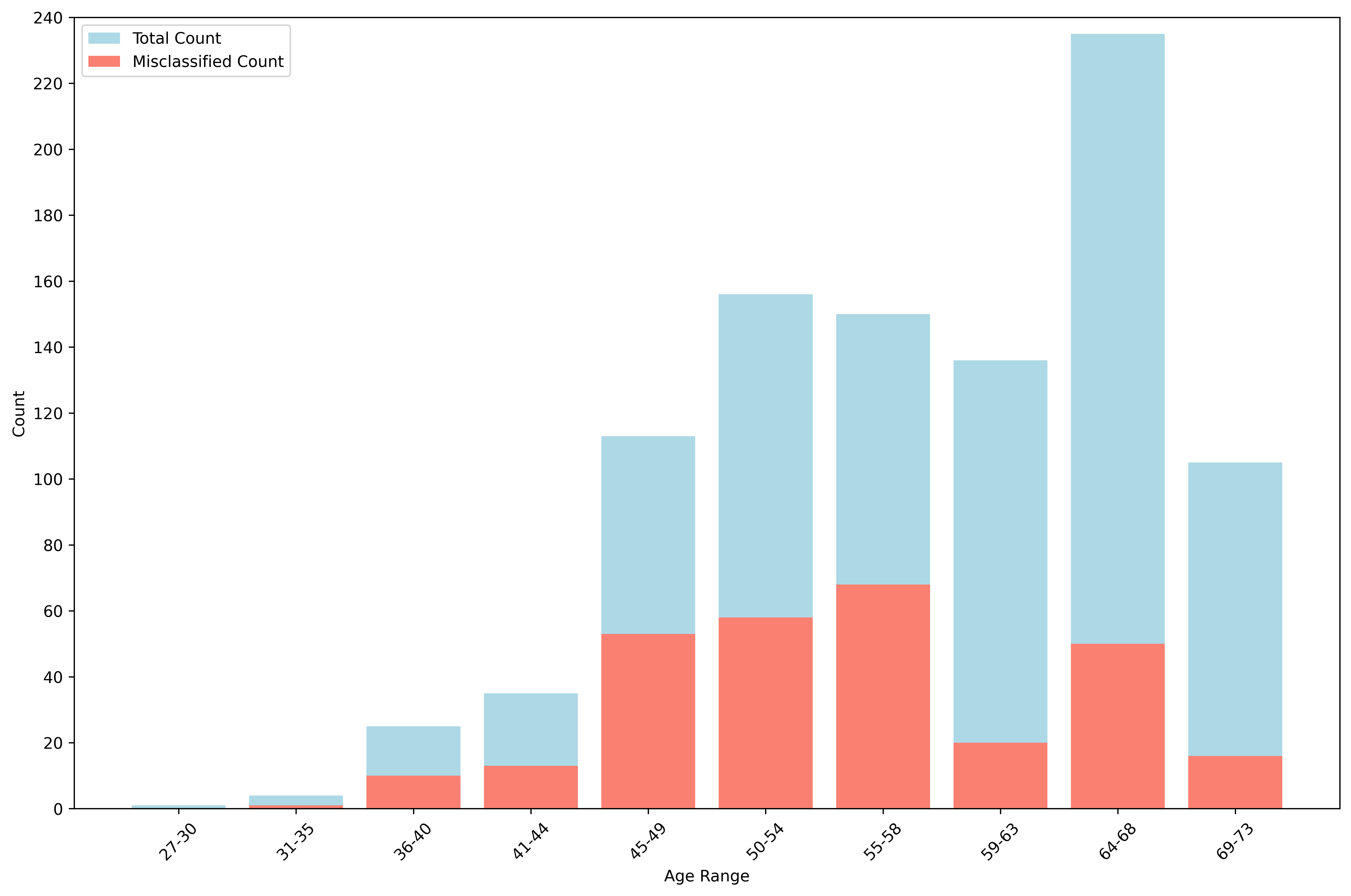

Supplement: Supplementary file 1 — Supplementary material [file mmc1.docx]
